# Thermoelectric Properties of Thin Films from Sorted Single-Walled Carbon Nanotubes

**DOI:** 10.3390/ma13173808

**Published:** 2020-08-28

**Authors:** Blazej Podlesny, Bogumila Kumanek, Angana Borah, Ryohei Yamaguchi, Tomohiro Shiraki, Tsuyohiko Fujigaya, Dawid Janas

**Affiliations:** 1 Department of Organic Chemistry, Bioorganic Chemistry and Biotechnology, Silesian University of Technology, B. Krzywoustego 4, 44-100 Gliwice, Poland; Blazej.Podlesny@polsl.pl (B.P.); Bogumila.Kumanek@polsl.pl (B.K.); 2 Department of Applied Chemistry, Graduate School of Engineering, Kyushu University, 744 Motooka, Nishi-ku, Fukuoka 819-0395, Japan; borah.angana.018@s.kyushu-u.ac.jp (A.B.); yamaguchi.ryohei.072@s.kyushu-u.ac.jp (R.Y.); shiraki.tomohiro.992@m.kyushu-u.ac.jp (T.S.); fujigaya.tsuyohiko.948@m.kyushu-u.ac.jp (T.F.); 3 International Institute for Carbon Neutral Energy Research (WPI-I2CNER), Kyushu University, Fukuoka 819-0395, Japan; 4 Center for Molecular Systems (CMS), Kyushu University, 744 Motooka, Nishi-ku, Fukuoka 819-0395, Japan

**Keywords:** carbon nanotubes, sorting, thermoelectric properties

## Abstract

Single-walled carbon nanotubes (SWCNTs) remain one of the most promising materials of our times. One of the goals is to implement semiconducting and metallic SWCNTs in photonics and microelectronics, respectively. In this work, we demonstrated how such materials could be obtained from the parent material by using the aqueous two-phase extraction method (ATPE) at a large scale. We also developed a dedicated process on how to harvest the SWCNTs from the polymer matrices used to form the biphasic system. The technique is beneficial as it isolates SWCNTs with high purity while simultaneously maintaining their surface intact. To validate the utility of the metallic and semiconducting SWCNTs obtained this way, we transformed them into thin free-standing films and characterized their thermoelectric properties.

## 1. Introduction

Carbon nanotubes (CNT) are one of the newest additions to the carbon nanomaterials family. Although several decades have passed since their (re)discovery [1,2], they are still among the most extensively studied materials, catching the attention of many research groups. Due to their exceptional electrical [3,4], mechanical [5], and optical [6] properties, CNTs have a high implementation potential for a spectrum of applications. One of the milestones was reached when scientists discovered that the way how a single layer of carbon atoms is assembled to form an single-walled carbon nanotubes (SWCNT) (quantified by the so-called chirality [7,8]) determines the properties [5,6,9,10,11] of the obtained material. For instance, chirality dictates whether SWCNT has a metallic or semiconductor character, which is vital to meet the SWCNT type with the right application [12]. Furthermore, the optical characteristics of semiconducting SWCNTs (s-SWCNTs) are also highly dependent on the chiral angle because the chiral angle controls the bandgap of the material. s-SWCNTs fluoresce, and the wavelength of emission is directly dependent on this parameter [13]. Thus, the application potential (or lack thereof) hinges on the ability to control the structure of SWCNTs.

Regardless of the manufacturing method, no selective synthesis strategy has been developed yet, which would give a monochiral product. Several techniques have been developed to differentiate SWCNTs after the synthesis to alleviate this problem [8]. Unfortunately, despite their merits, the methods which involve electrophoresis [14], chromatography [15,16], density gradient centrifugation [17,18] or selective solubilization with polymers [19,20,21], require the use of specialized equipment, expensive reagents or challenging to synthesize chemical compounds, which makes it hard to implement them on a large scale. Recently, the aqueous two-phase extraction (ATPE) method emerged as a potential antidote to this problem, which avoids the issues mentioned above. The foundations for this method were laid more than 125 years ago by Martinus Beijerinck, who first prepared aqueous biphasic systems, which were then put in common purification practice by Per-Åke Albertsson. He successfully adapted it for the extraction of biomolecules and cell elements [22] in the 1950s [23]. In general, ATPE is a type of extraction that uses preferential migration of compounds to one of two phases, which differ in hydrophilicity. The most commonly used ATPE extraction systems include polymer/polymer, polymer/salt, salt/short-chain alcohol, and polymer/ionic liquid [22] solution combinations. In the context of SWCNT separation, the most commonly used system consists of polyethylene glycol (PEG) as the top phase and dextran (DEX) or polyacrylamide (PAM) [24,25] as the bottom phase. Moreover, anionic surfactants such as sodium dodecyl sulfate (SDS), sodium cholate (SC), or sodium deoxycholate (DOC) are introduced to the biphasic system to direct the migration of SWCNTs between the phases. Such a routine enables the separation of SWCNTs due to length, diameter, conductivity, chirality, or handedness (left-handed/right-handed enantiomers) [26] after several steps. The ATPE variants are characterized by the ability to extract just one selected SWCNT type in one go [13,27] or they are conducted in a multi-step way to resolve all the components of the mixture. The reduction of steps in the former approach is possible through the use of chemical modulators, which introduced into a biphasic system, enhance the slight differences between SWCNT species. Modulators can be redox agents or chemical compounds having a substantial impact on the pH of the system [13,28,29], either of which facilitates the separation. At present, one of the pressing challenges of this field is to devise straightforward protocols of large-scale separation of SWCNTs by electrical character to open several potential applications for power engineering. So far, several ways of separation by the electrical nature [29,30,31] were developed to obtain metallic or semiconducting base materials for conducting wires [32] or thermogenerators [33], respectively.

Many types of equipment that surround us are characterized by low energy efficiency [34,35], which generates considerable amounts of waste heat. The solution to this problem may be to use thermoelectric devices capable of transforming thermal energy into electric currents based on the Seebeck effect [33]. Up until now, rare earth elements have mainly been used as thermoelectric materials, but they are often toxic, expensive, and available only in some areas of the world [36]. It may be beneficial to replace them with SWCNTs, which have already proven appreciable performance. What is more, they can be synthesized from renewable biological feedstocks available in almost any corner of the globe. In general, semiconducting SWCNTs have a high Seebeck coefficient [37,38], as opposed to metallic SWCNTs, due to the inherent differences of how they conduct electric charge. For example, Nakai and co-workers measured the Seebeck coefficient of unsorted SWCNTs to be 36 µV/K at room temperature. It was possible to increase the performance considerably upon isolation of a semiconducting fraction from the material with subsequent doping [37]. Exposure to nitric acid or oxygen to such SWCNTs species increased the Seebeck coefficients to 150 µV/K and 78 µV/K, respectively. At the same time, the Seebeck coefficient of the metallic fraction was just 14 µV/K. The results of subsequent studies confirmed this trend many times [39,40]. The higher the s-SWCNTs/m-SWCNTs ratio (up to a certain threshold), the more suitable is the material for thermopower generation [41]. That is because a perfect thermoelectric material should have both a high Seebeck coefficient and appreciable electrical conductivity while possibly the lowest thermal conductivity, which is challenging to realize due to the way these parameters are intertwined [42].

In this contribution, we present a fully scalable variant of the ATPE method, which allows obtaining semiconducting/metallic SWCNT materials in large amounts. By introducing perhydrol as a modulator of the partitioning process, we attained differentiation of large-diameter SWCNT due to the electrical character in one step. Then, we demonstrated how the separated semiconducting and metallic SWCNTs could be purified from PEG and DEX matrices by a thermal treatment. The successful nature of the proposed sorting and purification routines was validated by measuring electrical and thermoelectric properties of thin films prepared from the obtained material. Post-separated metallic SWCNTs revealed a significant increase in electrical conductivity, while the Seebeck coefficient of semiconducting material increased considerably.

## 2. Materials and Methods 

### 2.1. Materials and Reagents

Large-diameter SWCNTs (Tuball™, batch: 89-21082015, OCSiAl, Luxemburg), Dextran (DEX, average molecular weight of 70,000 Da, PanRecAppliChem, Germany), poly(ethylene glycol) (average molecular weight of 6000 Da, Alfa Aesar, Germany), sodium cholate (SC, PanRecAppliChem, Germany), sodium dodecyl sulfate (SDS, Sigma-Aldrich, St. Louis, MO, USA), methanol (Avantor, Poland), H_2_O_2_ (hydrogen peroxide, Sigma-Aldrich, USA) were all purchased from commercial sources. The chemical compounds had a p.a. class. The Millipore Elix 10 demineralization system (Milipore SAS, Molsheim, France) was used to prepare demineralized water for the study. The quality of the water was monitored by measuring its electrical conductivity.

### 2.2. Preparation of SWCNT Dispersions

SWCNT powder was introduced to a 2% aqueous solution of SC in such a way that the final concentration of nanocarbon material and volume of the dispersion were 1 mg/mL and 40 mL, respectively. The mixture was homogenized by ultrasound tip sonicator (Hielscher UP200St, Teltow, Germany) with a constant power input of 50 W for 1 h. The processed dispersion was kept in an ice bath to avoid the thermal desorption of the surfactant from the SWCNT surface during the process. The dispersion was centrifuged (Eppendorf Centrifuge 5804 R, Hamburg, Germany) at a constant temperature (18 °C) for 1 h at 15,314× *g* to remove the non-individualized SWCNTs and impurities. The upper 80% of supernatant after centrifugation was collected and used for experiments.

### 2.3. ATPE Protocol

**Figure 1:**
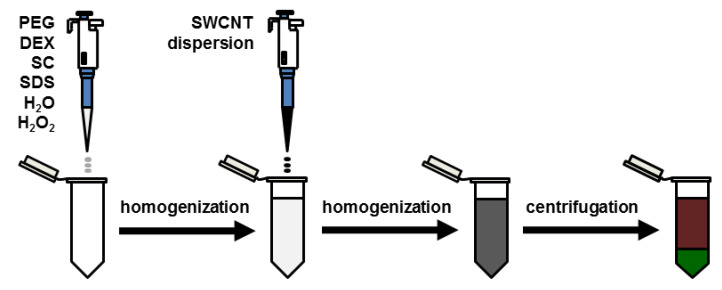
Sample processing methodology. The colors of the indicated phases correspond to the hues observed during the study.

Aqueous solutions of PEG (50 wt %), DEX (20 wt %), SC (10 wt %), SDS (10 wt %) and H_2_O_2_ (30 wt %) were put inside of a 50 mL Falcon centrifuge tube in the given order and vortexed for 15 s. Afterward, an appropriate amount of an SWCNT dispersion was added, and the mixture was homogenized again by vortexing. The obtained suspension was centrifuged for 3 min at a constant temperature (18 °C) at 2035× *g* centrifugal force to facilitate phase separation. After centrifugation, the mixture split into two phases—brown top and dark green bottom (Figure 1), which were collected immediately by pipetting.

### 2.4. Optical Characterization of the Sorted SWCNT Material

Optical absorption spectra were recorded in the wavelength range of 400–1100 nm using the Hitachi U-2910 spectrophotometer. Due to the high concentration of SWCNTs in the water medium, the samples were diluted with demineralized water for analysis with demineralized water. All spectra were normalized to the global minimum between 600 and 900 nm to compare the samples, which is a standard procedure [43].

### 2.5. Purification of the Samples from the Matrices

Dispersions of semiconducting and metallic SWCNTs embedded in PEG and DEX phases, respectively, were subjected to a multi-step purification protocol taking into account the differences in the chemical nature of these two polymer matrices. A detailed description of these routines is given below. 

#### 2.5.1. s-SWCNT in the PEG Phase

The dispersion of s-SWCNTs in the PEG phase was transferred to a round-bottomed flask equipped with a Liebig condenser and heating mantle. The material was then refluxed in water for 4 h to cause thermal desorption of surfactants and polymers from the SWCNT surface. As a consequence, the SWCNT material precipitated out at the bottom of the glassware. The progress of the desorption was monitored by visual inspection of the color, which gradually faded, eventually reaching transparency upon SWCNT sedimentation. Due to large amounts of various surfactants in the mixtures, foam was generated during the process, so glassware of appropriate capacity had to be employed to accommodate its volume. Once desorption was completed, the solid was separated by vacuum filtration (PTFE membrane, pore size: 0.22 µm; Fisherbrand, Ottawa, ON, Canada). The solid was treated with hot distilled water and then methanol. Next, the crude product was dispersed in water, which was facilitated by sonication. Resulting aqueous dispersion was refluxed for 6 h, and then left overnight to make the SWCNTs sediment. The supernatant was removed, fresh distilled water was added, and the heating-sedimentation-pipetting process was repeated twice. The material was subsequently boiled in HCl for 6 h at 60 °C to ensure the complete removal of PEG. Finally, s-SWCNTs were separated again by filtration (the same PTFE membrane type). Hot distilled water was used until the filtrate had neutral pH, and no foam could be discerned. The product was dried in a desiccator. 

#### 2.5.2. m-SWCNT in the DEX Phase

The dispersion of m-SWCNTs in the DEX phase was transferred to a round-bottomed flask equipped with a Liebig condenser and heating mantle. The material was then heated in acidic water (pH ~ 1) at 60 °C for 6 h to cause thermal desorption of surfactants and polymers from the SWCNT surface as well as to cause partial hydrolysis of DEX. The acidity of employed water and temperature should not be excessive so as not to cause DEX degradation. After the thermal treatment, the material was centrifuged for 10 min (18 °C, 15,314× *g*). The supernatant rich in residues of phase-forming components and surfactants was removed, and the SWCNT cake located at the bottom was collected. The crude product was then purified using filtration under reduced pressure (PTFE membrane, pore size: 0.22 µm; Fisherbrand, Ottawa, ON, Canada) with boiling distilled water. When the amount of foam was considerably reduced, the solid was moved to a round-bottom flask wherein concentrated HCl has been added. The mixture was kept in this state for 6 days to ensure the successful hydrolysis of DEX. The obtained precipitate was filtered (the same PTFE membrane type) by using solvents in the following order: hot distilled water, methanol and acetone to remove the contaminants. The product was dried in a desiccator overnight. 

### 2.6. SWCNT Films Preparation and Characterization

Firstly, 150 mg of dried SWCNT material was added to 80 mL of methanol and homogenized by ultrasonication. After obtaining uniform dispersion, it was filtered under reduced pressure to give a thin film. The SWCNT film collected on a membrane (PTFE membrane, pore size: 0.22 µm; Fisherbrand, Ottawa, ON, Canada) was peeled off the surface and used for the study. Then, 3 mm × 20 mm specimens were cut out from the produced films. Thermograms (TGA2 Mettler Toledo) were determined in the flow of air (30 mL/min) at a 10 °C/min heating rate from 30 to 1000 °C. A total of 1.0 mg of material was used for each measurement. SEM micrographs were acquired using Scanning Electron Microscope (Supra 35, Carl Zeiss, Oberkochen, Germany) at 5 kV acceleration voltage.

### 2.7. Characterization of Electrical and Thermoelectric Properties of Sorted SWCNT Films

The Seebeck coefficient and electrical conductivity of the samples were determined using a ZEM-3M system (ULVAC-Riko) under helium at reduced pressure (0.01 MPa) from ca. 30 to 100 °C. The measurements were conducted three times at least with different specimens. Conductance was converted to conductivity by taking into account the dimensions of the sample. The sample thickness was measured with a micrometer screw gauge (Electronic Universal IP54, Linear). Based on the obtained results of electrical conductivity and Seebeck coefficients, the Power Factors (PFs) were calculated using the following formula:

PF = S^2^σ (1)

where: PF—Power Factor [µW/m·K^2^], S—Seebeck coefficient [µV/K], σ—electrical conductivity [S/cm].

## 3. Results and Discussion

### 3.1. ATPE Protocol and Optical Characterization

**Figure 2:**
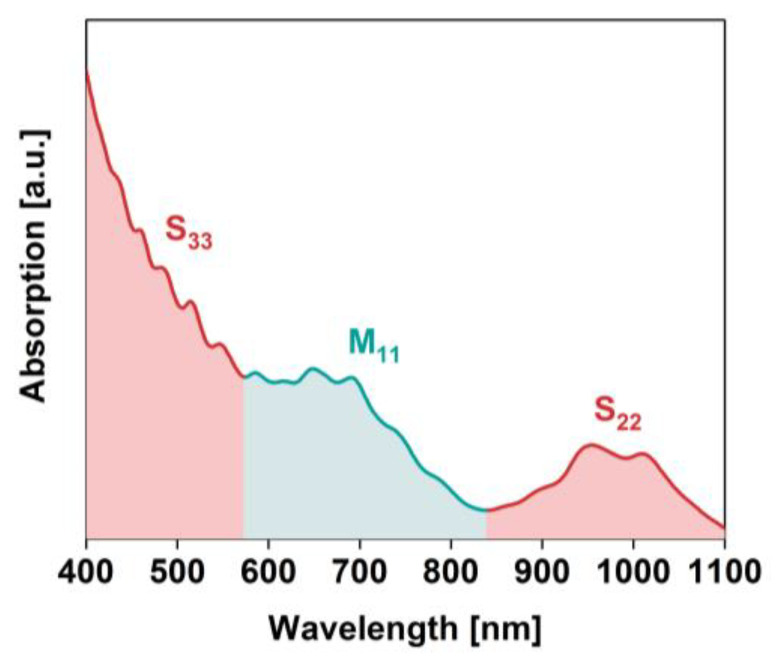
The absorption spectrum of starting material. Absorption ranges of semiconducting (S_22_ and S_33_) and metallic (M_11_) optical transition are indicated.

We used large-diameter SWCNTs of 1.8 ± 0.4 nm in diameter distribution. The material consisted of both metallic and semiconducting chiralities, which was confirmed by characterization of optical properties of the material by absorption spectroscopy (Figure 2). Analysis of the parent SWCNT dispersion showed optical transitions between the van Hove singularities of the Density of States from both metallic and semiconducting SWCNTs. Through light absorption, the charge carriers are promoted from the valence to the conduction band, which enables optical detection of SWCNTs. S_22_, S_33,_ and M_11_ ranges were located in the wavelength domains expected for large diameter species [44].

One-step adaptation of the ATPE method was employed here based on our previous work with hydrogen peroxide as a partitioning modulator [30]. Its mildly oxidizing properties facilitate the differentiation process, similarly to NaClO [29]. The electron transfer process upon the addition of H_2_O_2_ reorganizes hydration shells of semiconducting and metallic SWCNTs. It takes place dissimilarly depending on a SWCNT type due to the electronic differences, which drive the partitioning. 

**Figure 3:**
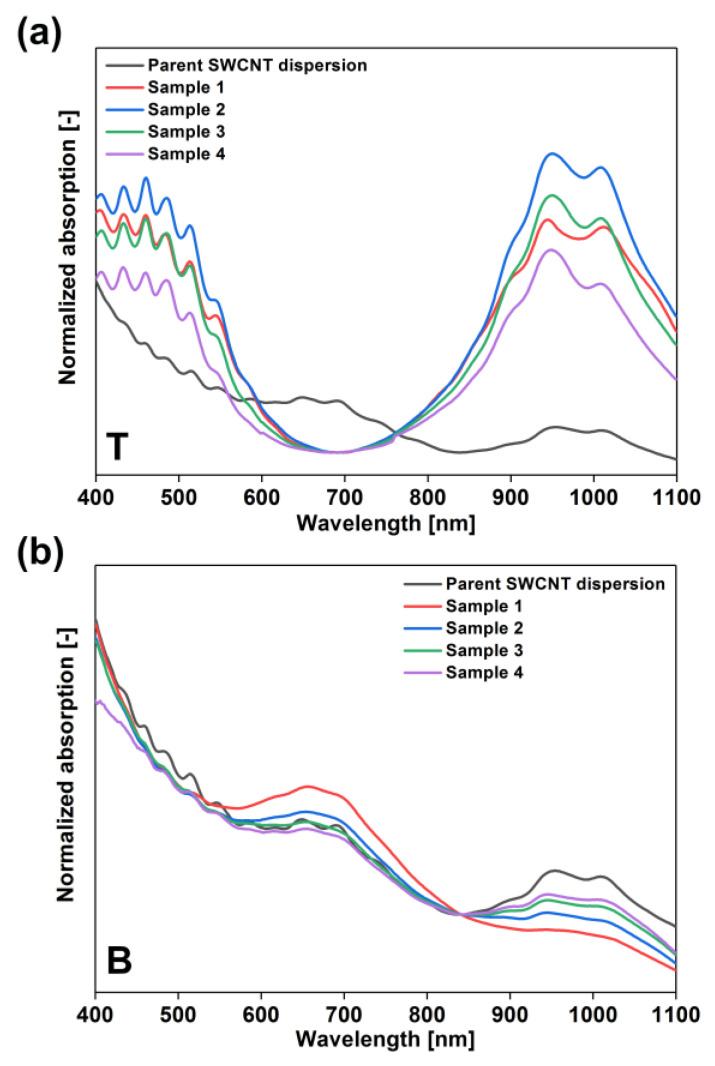
Absorption spectra of (**a**) top phases (PEG) and (**b**) bottom phases (DEX) of Samples 1–4 collected after ATPE separation at a fourfold higher concentration with varying contents of the H_2_O_2_ modulator introduced at the expense of water. In both cases, the optical absorption spectrum of the parent dispersion is given as a reference.

Most of the ATPE experiments reported so far involve separation of SWCNTs at a rather low concentration [26], therefore we decided to target this problem. Although working under a low-concentration regime enables high-resolution separation, it is problematic from the practical point of view because it increases the unit separation costs. It is essential to devise high throughput routines, which could handle much more SWCNTs per one ATPE separation experiment to increase the application potential of the sorted material. We initiated this study by running the separation routine at a fourfold higher SWCNT concentration than previously reported (Table 1, Figure 3). 

The amount of introduced H_2_O_2_ into the biphasic system was found to have a strong influence on the course of partitioning. Firstly, clearly the presence of H_2_O_2_ was beneficial as semiconducting and metallic SWCNTs were detected in the top and bottom phases, respectively, upon its addition. 

Nevertheless, the first results showed that, at a low content of SWCNTs (φ_CNT_ = 4.90) and high amounts of H_2_O_2_ (φ_H2O2_ = 6.54), the differentiation was more selective (Sample 1). The metallic fraction in the bottom phase had only a small amount of semiconducting species judging by the barely visible peak corresponding to S_22_ transitions between 850 and 1100 nm. The composition of the top phase was also highly enriched with semiconducting SWCNTs and no metallic SWCNTs were detected therein. As the SWCNT content was quadrupled (φ_CNT_ = 19.61), the top fractions were still of high semiconducting purity, but the metallic bottom phases suffered from contamination. 

We first discuss the impact of the amount of added H_2_O_2_ on the composition of the top phases. A non-monotonic relation between the relative amount of H_2_O_2_ and the degree of partitioning was observed in these experiments. The low φ_H2O2_ volume fractions of 1.31 and 2.61 (Samples 2 and 3, respectively) afforded a high content of semiconducting SWCNTs in the top phase. The intensity of both S_22_ and S_33_ exceeded that of Sample 1. The maximum semiconducting purity was attained at the lowest volume of introduced H_2_O_2_ (Sample 2, φ_H2O2_ = 1.31). Then, a further increase in the volume of H_2_O_2_ resulted in the gradual deterioration of the partitioning. The top fraction of Sample 3 (φ_H2O2_ = 2.61) had a lower content of semiconducting SWCNTs than Sample 2, but still higher than Sample 1. However, upon eventually reaching the φ_H2O2_ of 5.23 (Sample 4) the purity was the lowest among all investigated dispersions. Based on the obtained data, the optimum conditions for isolation of the highest amount of semiconducting SWCNTs are φ_H2O2_ = 1.31 and φ_CNT_ = 19.61 corresponding to the conditions used to prepare Sample 2. 

With regard to the composition of the bottom phases, only the parameters used for the preparation of Sample 1 made it rich in metallic species. In all the remaining fractions (Samples 2–4), the contamination of the metallic portion with semiconducting SWCNTs was evident. It can be concluded that, at a relatively high amount of introduced SWCNTs (Samples 2–4), high-throughput isolation of semiconducting SWCNTs in the top phase is achieved at the expense of the purity of bottom samples. Under a high SWCNT concentration regime, the smallest contamination was determined again in the case of Sample 2. In such a case, the intensity of the band of S_22_ transitions was the lowest. Therefore, we concluded that the optimum amount of added H_2_O_2_ at a fourfold higher concentration of SWCNTs corresponds to φ_H2O2_ of 1.31 (Sample 2).

**Figure 4:**
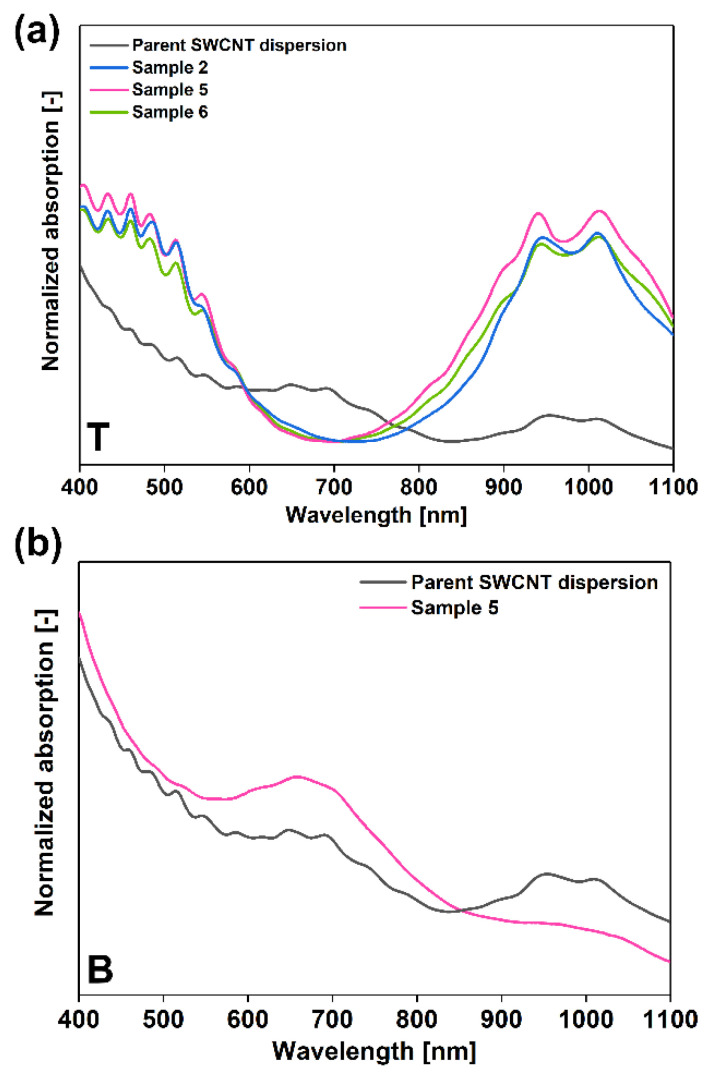
Absorption spectra of (**a**) top phases (PEG) of Samples 2, 5 and 6 and (**b**) bottom phase (DEX) of Sample 5 collected after ATPE separation at a selected concentration of H_2_O_2_ with varying contents of the introduced SWCNT dispersion. In both cases, the optical absorption spectrum of the parent dispersion is provided as a reference.

In the next step, we wanted to optimize the relative amount of H_2_O_2_ to SWCNTs to enhance the purity of the bottom metallic fraction. We decided to deal with this challenge by lowering the amount of introduced SWCNT dispersion for sorting. This was found necessary, as the quadruple concentration of SWCNTs (φ_CNT_ = 19.61) as compared to earlier work (φ_CNT_ = 4.90) [30] was found excessive from the practical point of view. Such a high content of SWCNTs did not enable us to see the interface between the phases, which could lead to mistakes in pipetting, so it had to be lowered. We explored two additional separation conditions with φ_CNT_ = 14.71 and φ_CNT_ = 9.80 (Table 2, Figure 4), which correspond to three- and two-fold higher SWCNT concentrations than in our previous study. 

**Figure 5:**
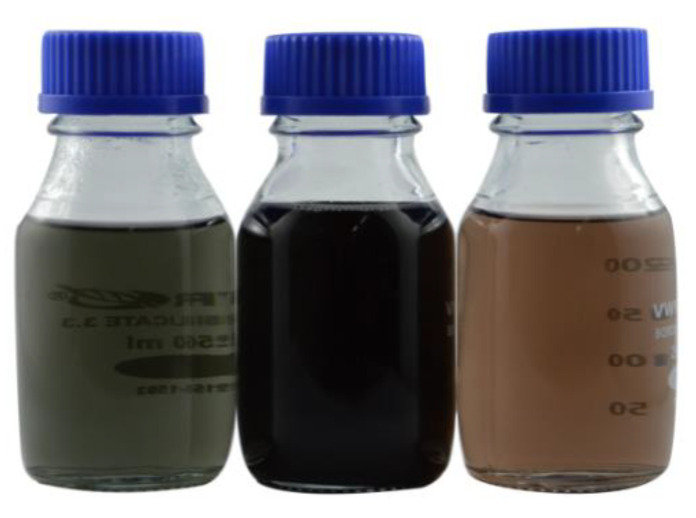
The photograph of parent SWCNT dispersion (**middle**) and collected post-separation phases: top (semiconducting, **right**) and bottom (metallic, **left**). Top and bottom phases were diluted with H_2_O (top in ratios 1:4, bottom in ratios 1:40) for a clear illustration of colors.

By decreasing the SWCNT amount to the former concentration (φ_CNT_ = 14.71), the interface already became visible, and the phases could be separated from each other with ease. Moreover, analysis of the absorption spectra of the sample showed that, by a small decrease in the amount of processed SWCNTs, the content of semiconducting species is even slightly higher in the top phase (Sample 5, Figure 4a). Further decrease of the amount of separated SWCNTs to φ_CNT_ = 9.80 (Sample 6) was found impractical. Not only did it decrease the throughput of the separation system, but the amount of isolated semiconducting SWCNTs in the top was also reduced. Interestingly, under the separation conditions of Sample 5, the purity of the bottom phase containing metallics was high, judging on the predominance of the M_11_ feature in the absorption spectrum (Figure 4b). We managed to optimize the conditions using three-fold higher concentration than in our previous report. Therefore, we decided to scale the process up to generate a sufficient amount of material to make free-standing sorted SWCNT films and analyze their thermoelectric properties (Figure 5). 

To obtain several hundred milligrams of pure large-diameter metallic or semiconducting SWCNT, we used the optimized parameters determined for Sample 5. To our delight, the collected fractions showed expected colors, and their intensity was high. In fact, for the photographs, SWCNT dispersions had to be diluted by the specified amount of water to reduce the light absorption and hence reveal the colors. The top metallic phases and the bottom semiconducting species had burgundy and dark-green colors, respectively.

### 3.2. Purification

**Figure 6:**
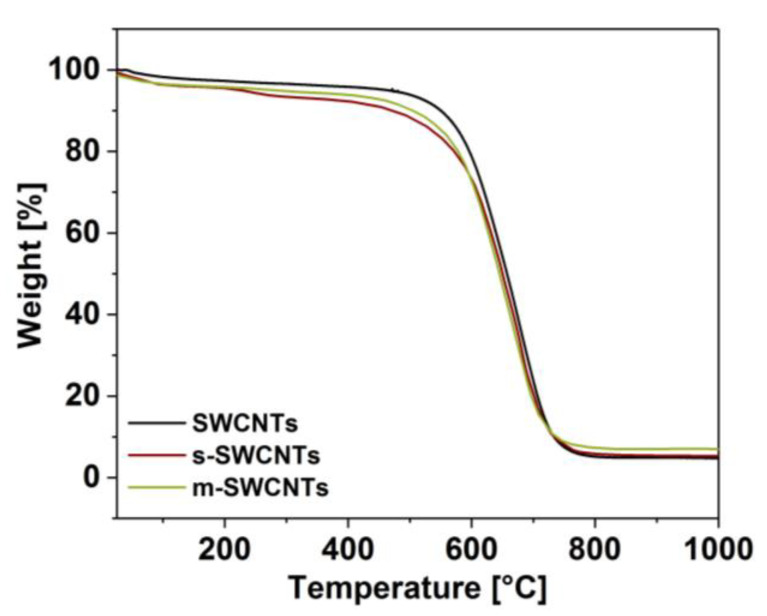
Thermograms of separated and purified s-SWCNTs and m-SWCNTs. Raw SWCNT material is given as a reference.

The sorted SWCNT dispersions had to be isolated from PEG and DEX matrices to make the material suitable for the study. The obtained SWCNTs as dry solids, after the described purification methodology, were subjected to thermogravimetric analysis (Figure 6). This step served a dual purpose. Firstly, this was to probe whether the proposed routine enables the generation of material free of contamination. Secondly, it was essential to check if the processing does not deteriorate the material. We observed that the obtained material is both pure and free of additional defects. The thermograms of s-SWCNTs and m-SWCNTs very much resembled that of the starting material. Hence, we concluded that the material is appropriate for the preparation of films to study them from the thermoelectric perspective. Regarding the morphology, thin films produced from these materials did not reveal any changes as compared with the reference (Figure S1). The microstructure of the films observed by SEM was virtually the same regardless of whether SWCNTs were sorted or not, which validates the non-destructive character of the reported separation-purification routine. 

### 3.3. Characterization of Thermoelectric Properties

**Figure 7:**
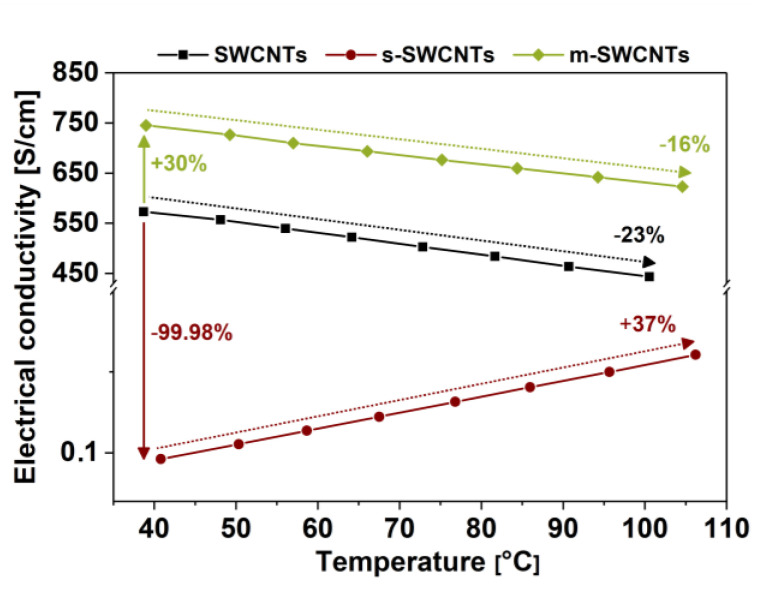
Electrical conductivity of thin films from unsorted, metallic, and semiconducting SWCNTs. Horizontal arrows compare the increase or decrease of the initial electrical conductivity of metallic and semiconducting films as related to the parent material. The arrows, which span across selected temperature ranges, on the other hand, compare the electrical conductivity of the respective SWCNT networks as related to the initial value of electrical conductivity recorded at the starting temperature.

**Figure 8:**
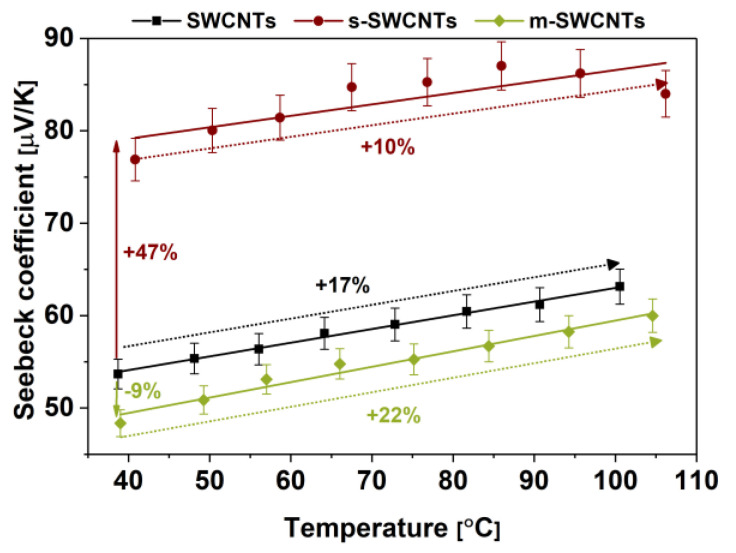
Seebeck coefficients of thin films from unsorted, metallic, and semiconducting SWCNTs. Horizontal arrows compare the increase or decrease of initial Seebeck coefficients of metallic and semiconducting films as related to the parent material. The arrows, which span across selected temperature ranges, on the other hand, compare the Seebeck coefficients of the respective SWCNT networks as related to the initial value of electrical Seebeck coefficients recorded at the starting temperature.

Our results show that, before the separation, the unsorted SWCNT material is characterized by both electrical conductivity (Figure 7) and the Seebeck coefficient (Figure 8) at the levels between that of predominantly metallic and semiconducting fractions. Such results are in line with our predictions and demonstrate successfully conducted differentiation. The mixture contains a metallic fraction that guarantees high electrical conductivity and the semiconductor fraction that is crucial for appreciable thermoelectric properties. The division of SWCNTs into these two types enabled us to study their behavior.

For m-SWCNTs, we recorded an increase in electrical conductivity of about 30% as compared to the unsorted material, and its value was 750 S/cm vs. 573 S/cm (Figure 7). On the other hand, for s-SWCNTs film, a very significant decrease in conductivity was recorded. The separation resulted in a reduction of electrical conductivity down to 0.10 S/cm. Such a sharp decrease in electrical conductivity upon isolation of an s-SWCNT fraction was to be expected because these SWCNTs conduct current much less facilely than m-SWCNTs. What is more, as the temperature of the films was increased, we observed two opposite effects. The conductivity of m-SWCNT film decreased with temperature, whereas it raised for the s-SWCNT analogous film. The former can be explained by increased scattering rate in metallic conductors, which is caused by the increased probability of collision of charge carriers with phonons as the temperature is elevated [45]. That is why we experienced ca. 16% decrease in electrical conductivity as the temperature of the m-SWCNT film was increased beyond 100 °C. What regards the behavior of s-SWCNTs, as the temperature is elevated, thermal energy facilitates the promotion of the charge carriers to the conduction band [46], which makes the material more conductive. Hence, a 37% improvement was observed for s-SWCNTs when the SWCNT network operated at high temperatures. 

Then, we moved on to the characterization of Seebeck coefficients of obtained material (Figure 8). The maximum Seebeck coefficient was recorded for s-SWCNTs and amounted to 87 μV/K at 100 °C. 

It is almost a 50% increase as compared with the unsorted materials. On the other hand, the Seebeck coefficients for films made from unsorted and m-SWCNTs had a similar performance of 63 and 60 μV/K, respectively. We recorded a slight decrease (9%) in the Seebeck coefficients for networks made of m-SWCNTs similarly (as compared with that of the unsorted material). It is to be expected because metallic SWCNTs are less suitable for thermoelectrics than semiconducting materials^37^. Besides, we have observed that, as the temperature increases, the material’s ability to convert thermal energy into electrical energy is also enhanced. An increase of 22%, 17%, and 10% was recorded for m-SWCNTs, unsorted SWCNTs, and s-SWCNTs, respectively, upon reaching ca. 100 °C, which mimicked the behavior established earlier for MWCNTs [47]. The slope of all of these trend lines is almost the same, which shows that, regardless of the type of SWCNTs constituting the film, the response of the Seebeck coefficient to temperature is roughly the same. 

**Figure 9:**
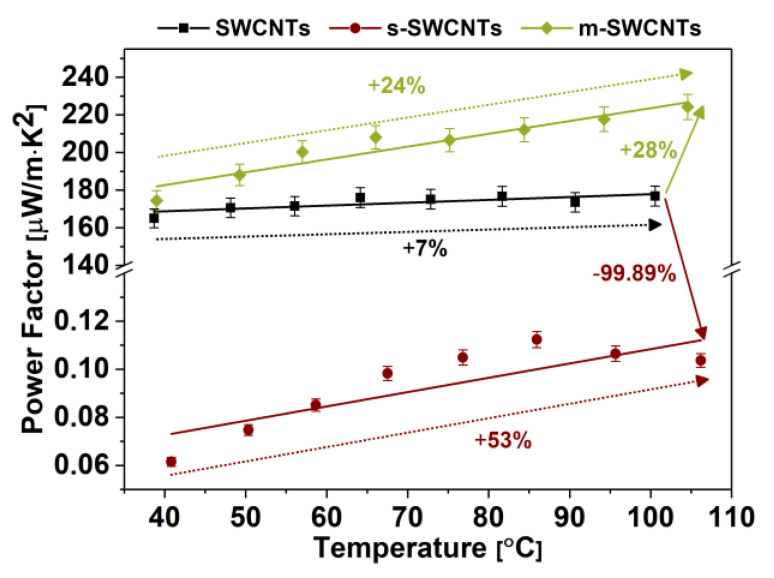
Power Factors (PFs) of thin films from unsorted, metallic, and semiconducting SWCNTs. Horizontal arrows compare the increase or decrease of initial Power Factor of metallic and semiconducting films as related to the parent material. The arrows, which span across selected temperature ranges, on the other hand, compare the Power Factors of the respective SWCNT networks as related to the initial value of Power Factor recorded at the starting temperature.

The factor that informs us about the suitability of a given material for thermoelectric applications and allows us to compare it with other competing solutions is Power Factor (PF). It takes into account the Seebeck coefficient and the electrical conductivity (neglecting the impact of thermal conductivity, which may be challenging to determine at times). Figure 9 compares the PF results obtained for the films made from unsorted SWCNTs as well as those of predominantly metallic and semiconducting character. 

Due to the meager electrical conductivity value that characterizes s-SWCNTs, the PF value for that material is unsatisfactory (0.073 μW/m·K^2^). A high value of the Seebeck coefficient under these conditions could not compensate for the low carrier concentration to reach an appropriate PF [33]. In the case of the films made of unsorted SWCNTs and m-SWCNTs, the PF values at near room temperature are similar (165 μW/m·K^2^ and 175 μW/m·K^2^, respectively). These PF are three orders of magnitude higher than the PF value determined for s-SWCNTs. As the temperature rises, the PF value for s-SWCNTs, m-SWCNTs, and unsorted SWCNTs increases by 53%, 24%, and 7%, respectively. Tracking the influence of temperature on PF is more difficult to perceive because it is linearly dependent on the electrical conductivity, whereas the Seebeck coefficient makes a quadratic impact. In the case of s-SWCNTs, the relative change was the largest because both electrical conductivity and Seebeck coefficient increased with temperature. On the other hand, although the conductivity of m-SWCNTs decreased with temperature, this effect was overpowered by the increase in the Seebeck coefficient. The results of the study indicate that, based on the results of PF determination, the sample type with the highest thermoelectric performance was made from metallic SWCNTs despite what one could expect due to a more proper amount of charge carriers. 

## 4. Conclusions

In summary, we presented a practical methodology on how to separate large-diameter SWCNTs by the ATPE method (metallic/semiconducting) and isolate the obtained material from the polymer matrices. Harvested SWCNTs were then transformed into thin free-standing films, which were subjected to the characterization of their electrical and thermoelectric properties. The results showed that the proposed purification strategy is advantageous. Firstly, it does not leave any residue in the SWCNT material, which could interfere when studying the material. Secondly, the facile approach left the SWCNTs after the processing intact as their thermograms very much resembled that of the starting material. The results of characterization demonstrated considerable differences between the electrical and thermoelectric properties of semiconducting and metallic SWCNTs. Relatively high Power Factor values resulted from the application of high-quality large-diameter SWCNTs as the starting material, which ensured appreciable electrical conductivity. One also has to keep in mind that large-diameter semiconducting SWCNTs have negligible bandgaps, and thus charge carriers in them can easily be thermally excited. 

This study illustrates that it is of utmost importance not only to synthesize materials and nanomaterials of defined structure and properties but also to develop simple strategies of their processing. (Nano) materials are commonly generated from complex mixtures of components, many of which persist in the final product. In turn, the presence of adulterants makes it very challenging to elucidate underlying phenomena. This problem is particularly problematic in the case of nanomaterials. What is also common is that it makes the results much less reproducible. Therefore, an appropriate process design has to be executed to alleviate these issues. 

## Figures and Tables

**Table 1:** Compositions of aqueous two-phase extraction method (ATPE) systems for separation of single-walled carbon nanotubes (SWCNTs) at high concentrations with varying contents of the H_2_O_2_ modulator.

Compound	Volume Fraction, *φ_i_* [-]			
	Sample 1 (Previous Work [30])	Sample 2	Sample 3	Sample 4
PEG, 50 wt %, aq.	11.76	11.76	11.76	11.76
DEX, 20 wt %, aq.	29.41	29.41	29.41	29.41
SC, 10 wt %, aq.	7.84	7.84	7.84	7.84
SDS, 10 wt %, aq.	3.92	3.92	3.92	3.92
H_2_O_2_, 30 wt %, aq.	6.54	1.31	2.61	5.23
SWCNTs, 0.1 wt %, 2 wt % SC aq.	4.90	19.61	19.61	19.61
H_2_O	35.62	26.14	24.84	22.22

**Table 2:** Compositions of ATPE systems for separation of SWCNTs at a selected concentration of the H_2_O_2_ modulator with varying contents of SWCNT dispersion.

Compound	Volume Fraction, *φ_i_* [-]		
	Sample 2	Sample 5	Sample 6
PEG, 50 wt %, aq.	11.76	11.76	11.76
DEX, 20 wt %, aq.	29.41	29.41	29.41
SC, 10 wt %, aq.	7.84	7.84	7.84
SDS, 10 wt %, aq.	3.92	3.92	3.92
H_2_O_2_, 30 wt %, aq.	1.31	1.31	1.31
SWCNTs, 0.1 wt %, 2 wt % SC aq.	19.61	14.71	9.80
H_2_O	26.14	31.05	35.95

## Supplementary Materials

The following are available online at https://www.mdpi.com/1996-1944/13/17/3808/s1, Figure S1: SEM micrographs of thin films from unsorted, metallic, and semiconducting SWCNTs.
