# Supplementary material for: Thermoelectric Properties of Thin Films from Sorted Single-Walled Carbon Nanotubes
Source: Materials (Basel). 2020 Aug 28;13(17):3808. doi: 10.3390/ma13173808 (PMC7504438; doi:10.3390/ma13173808)
Supplement: Supplementary file 1 [file materials-13-03808-s001.pdf]

materials

Supplementary Information

Thermoelectric Properties of Thin Films from Sorted Single-Walled Carbon Nanotubes

Blazej Podlesny [1], Bogumila Kumanek [1], Angana Borah [2], Ryohei Yamaguchi [2], Tomohiro Shiraki [2,3], Tsuyohiko Fujigaya [2,3,4] and Dawid Janas [1,*]

1 Department of Organic Chemistry, Bioorganic Chemistry and Biotechnology, Silesian University of Technology, B. Krzywoustego 4, 44-100 Gliwice, Poland; Blazej.Podlesny@polsl.pl (B.P.); Bogumila.Kumanek@polsl.pl (B.K.)
2 Department of Applied Chemistry, Graduate School of Engineering, Kyushu University, 744 Motooka, Nishi-ku, Fukuoka 819-0395, Japan; borah.angana.018@s.kyushu-u.ac.jp (A.B.); yamaguchi.ryohei.072@s.kyushu-u.ac.jp (R.Y.); shiraki.tomohiro.992@m.kyushu-u.ac.jp (T.S.); fujigaya.tsuyohiko.948@m.kyushu-u.ac.jp (T.F.)
3 International Institute for Carbon Neutral Energy Research (WPI-I2CNER), Kyushu University, Fukuoka 819-0395, Japan
4 Center for Molecular Systems (CMS), Kyushu University, 744 Motooka, Nishi-ku, Fukuoka 819-0395, Japan
* Correspondence: Dawid.Janas@polsl.pl; Tel.: +48-32-237-1082

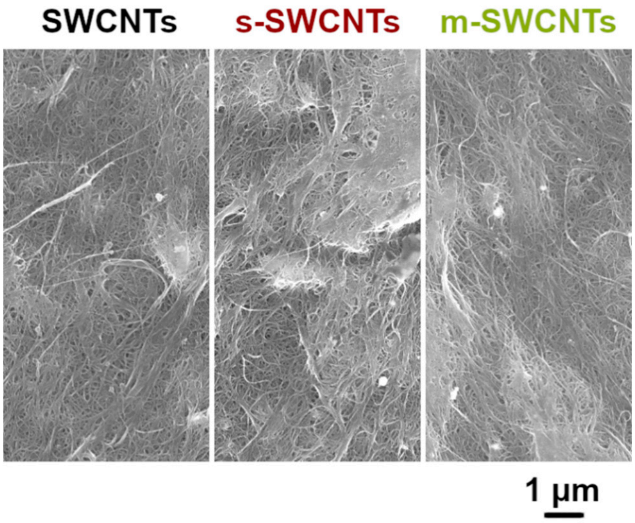

Figure S1 SEM micrographs of thin films from unsorted, metallic, and semiconducting SWCNTs.
